# Training modalities in robot-mediated upper limb rehabilitation in stroke: a framework for classification based on a systematic review

**DOI:** 10.1186/1743-0003-11-111

**Published:** 2014-07-10

**Authors:** Angelo Basteris, Sharon M Nijenhuis, Arno HA Stienen, Jaap H Buurke, Gerdienke B Prange, Farshid Amirabdollahian

**Affiliations:** 1 Adaptive Systems Research Group, School of Computer Science, University of Hertfordshire, College Lane, AL95HX Hatfield, United Kingdom; 2 Roessingh Research and Development, Roessinghsbleekweg 33b, 7522 AH Enschede, The Netherlands; 3 Laboratory of Biomechanical Engineering, University of Twente, Drienerlolaan 5, 7522 NB Enschede, The Netherlands; 4 Department of Biomedical Signals and Systems, University of Twente, Drienerlolaan 5, 7522 NB Enschede, The Netherlands; 5 Physical Therapy and Human Movement Sciences, Northwestern University, 645 N Michigan Ave Apt 1100, 60611 Chicago, IL, USA

**Keywords:** Therapeutic interaction, Robotics, Stroke, Neurorehabilitation, Arm, Wrist, Hand, Upper extremity

## Abstract

Robot-mediated post-stroke therapy for the upper-extremity dates back to the 1990s. Since then, a number of robotic devices have become commercially available. There is clear evidence that robotic interventions improve upper limb motor scores and strength, but these improvements are often not transferred to performance of activities of daily living. We wish to better understand why. Our systematic review of 74 papers focuses on the targeted stage of recovery, the part of the limb trained, the different modalities used, and the effectiveness of each. The review shows that most of the studies so far focus on training of the proximal arm for chronic stroke patients. About the training modalities, studies typically refer to active, active-assisted and passive interaction. Robot-therapy in active assisted mode was associated with consistent improvements in arm function. More specifically, the use of HRI features stressing active contribution by the patient, such as EMG-modulated forces or a pushing force in combination with spring-damper guidance, may be beneficial.

Our work also highlights that current literature frequently lacks information regarding the mechanism about the physical human-robot interaction (HRI). It is often unclear how the different modalities are implemented by different research groups (using different robots and platforms). In order to have a better and more reliable evidence of usefulness for these technologies, it is recommended that the HRI is better described and documented so that work of various teams can be considered in the same group and categories, allowing to infer for more suitable approaches. We propose a framework for categorisation of HRI modalities and features that will allow comparing their therapeutic benefits.

## Introduction

Stroke is one of the most common causes of adult disabilities. In the United States, approximately 795,000 individuals experience a new or recurrent stroke each year, and the prevalence is estimated at 7,000,000 Americans over 20 years of age [1]. In Europe, the annual stroke incidence rates are 141.3 per 100,000 in men, and 94.6 in women [2]. It is expected that the burden of stroke will increase considerably in the next few years [3]. The high incidence, in combination with an aging society, indicates future increases in incidence, with a strong impact on healthcare services and related costs.

Impairments after stroke can result in a variety of sensory, motor, cognitive and psychological symptoms. The most common and widely recognised impairments after stroke are motor impairments, in most cases affecting the control of movement of the face, arm, and leg on one side of the body, termed as hemiparesis. Common problems in motor function after hemiparetic stroke are muscle weakness [4-6], spasticity [4-6], increased reflexes [4], loss of coordination [4,7] and apraxia [4]. Besides, patients may show abnormal muscle co-activation, implicated in stereotyped movement patterns, which is also known as ‘flexion synergy’ and ‘extension synergy’ [8,9]. Concerning the upper extremity, impaired arm and hand function contributes considerably to limitations in the ability to perform activities of daily living (ADL). One of the goals of post stroke rehabilitation is to regain arm and hand function, since this is essential to perform activities of daily living independently.

### Stroke rehabilitation

Stroke rehabilitation is often described as a process of active motor relearning that starts within the first few days after stroke. Recovery is characterized by a high inter-individual variability, and it occurs in different processes. Some of the first events following nervous system injury are recovery due to restitution of non-infarcted penumbral areas, reduction of oedema around the lesion, and resolution of diaschisis [10-12], and comprise spontaneous neurological recovery. A longer term mechanism involved in neurological recovery is neuroplasticity, caused by anatomical and functional reorganisation of the central nervous system. Additionally, motor recovery after stroke may occur through compensational strategies. Compensation is defined as behavioural substitution, which means that alternative behavioural strategies are adopted to complete a task. In other words, function will be achieved through alternative processes, instead of using processes of ‘true recovery’ alone [11-13].

### Treatment approaches

Many treatment approaches have been developed to aid motor recovery after stroke. These interventions are different in their approach to achieve functional gains. For instance, in the 1950s and 1960s, the so-called neurofacilitation approaches were developed. From these approaches based on neurophysiological knowledge and theories, the Bobath Concept, or neurodevelopmental treatment (NDT), is the most used approach in Europe [14-16]. This approach focuses on normalizing muscle tone and movement patterns, guided by a therapist using specific treatment techniques, in order to improve recovery of the hemiparetic side. Gradually, focus shifted towards the motor learning, or relearning approach [17]. Others have referred to these methods as the task-oriented approach. These new methods of clinical practice are based on the notion that active practice of context-specific motor tasks with suitable feedback will support learning and motor recovery [11,17].

Overall, there is a lack of convincing evidence to support that any physiotherapy approach is more effective in recovery than any other approach [15,16,18]. However, in a review of Langhorne et al. [19], it is stated that some treatments do show promise for improving motor function, particularly those that focus on high-intensity and repetitive task-specific practice. Moreover, research into motor relearning and cortical reorganisation after stroke has showed a neurophysiologic basis for important aspects that stimulate restoration of arm function [20-23]. These important aspects of rehabilitation training involve functional exercises, with high intensity, and with active contribution of the patient in a motivating environment.

### Rehabilitation robotics

Robot-mediated therapy for the upper limb of stroke survivors dates back to the 1990s. Since then a number of robotic devices have become commercially available to clinics and hospitals, for example the InMotion Arm Robot (Interactive Motion Technologies Inc., USA, also known as MIT-Manus) and the Armeo Power (Hocoma, Switzerland). Robotic devices can provide high-intensity, repetitive, task-specific, interactive training. Typically, such robots deliver forces to the paretic limb of the subject while practicing multi-joint gross movements of the arm. Most of the robotic devices applied in clinical trials or clinical practice offer the possibility of choosing among four modalities for training: active, active-assisted, passive and resistive. These terms relate to conventional therapy modes used in clinical practice and refer to subject’s status during interaction. Passive training for example refers to subject-passive/robot-active training such as in continuous passive motion (CPM) devices. The choice of modality (−ies) in each protocol is ultimately made by researchers/therapists.

There is evidence that robotic interventions improve upper limb motor scores and strength [24-26], but these improvements are often not transferred to performance of activities of daily living (ADL). These findings are shared among the most recent studies, including the largest randomised controlled trial related to robot-therapy to date [27]. A possible reason for a limited transfer of motor gains to ADL is that the earlier studies on robot-mediated therapy have only focused on the proximal joints of the arm, while integration of distal with proximal arm training has been recognized as essential to enhance functional gains [28,29].

Another issue involved in the limited transfer of motor gains to ADL improvements in robot-mediated therapy research may relate to the large variety of devices and protocols applied across clinical trials. Lumping together many devices and protocols does not provide knowledge of the effectiveness of individual components, such as which of the available therapeutic modalities result in the largest effect [25]. Consequently, in literature there has been a transition towards reviews focusing on selective aspects of robot-mediated therapy, rather than its overall effectiveness [30-33].

A major step in that direction is the description of different control and interaction strategies for robotic movement training. In a non-systematic review, Marshal-Crespo et al. [34] collected a set of over 100 studies involving both upper and lower limb rehabilitation. They made a first distinction between assistive, challenging and haptic-simulating control strategies. They also described assistive impedance-based controllers, counterbalancing, EMG-based and performance-adapted assistance. Furthermore, they highlighted the need for trials comparing different interaction modalities. However, that review only included articles up to the year 2008 while much more new information has become available in the recent years.

Loureiro et al. [35] described 16 end-effector and 12 exoskeleton therapy systems in terms of joints involved, degrees of freedom and movements performed [35]. However, this non-systematic review did not report about the effects of the interventions or identify the interaction or control strategies used. Specifically focusing on training of the hand, Balasubramanian et al. [36] identified 30 devices for hand function and described them in terms of degrees of freedom, movements allowed, range of motion, maximum force (torque) and instrumentation. Among these devices, eight showed an improvement in functional use of the affected hand, in terms of increased scores on the Action Research Arm Test, Box and Block test or Wolf Motor Function Test [36]. Understanding and specifically targeting mechanisms underlying recovery of the entire upper limb after stroke is essential to maximize improvements on function or even activity level.

This literature study works towards clarifying the definitions adopted in robotic control and interaction strategies for the hemiparetic upper extremity (including both proximal and distal arm segments), and identifying the most promising approaches. The objective of this systematic review is to explore and identify the human robot interaction mechanisms used by different studies, based on the information provided in literature. We propose a framework to support future categorisation of various modalities of human-robot interaction and identify a number of features related to how such strategies are implemented. In addition, we will compare clinical outcome in terms of arm function and activity improvements associated with those interactions, which allows us to identify the most promising types of human robot interactions.

## Methods

We conducted a systematic literature search on PubMed with keywords including *stroke*, *robot* and *arm, upper limb, shoulder, elbow, wrist* or *hand*. Detailed information about the search strategy is provided in Additional file 1: Appendix 1. We included full journal papers written in English about robotic training of (any part of) the upper limb. These included either uncontrolled (pre-post design) or (randomised) controlled trials, in which a group of at least four subjects received robot-mediated training. In addition, training outcome must be statistically evaluated (either pre- or post-treatment for the single group or a difference between groups). In cases where results from the same subjects were presented (partially) in other studies (e.g. a pilot study and the definitive protocol) we retained the study with the largest number of participants or the most recent study, if the number of participants were the same. Also, we discarded those studies where other interventions were applied during robot-mediated exercise (e.g. functional electrical stimulation). Two independent reviewers (AB and SN) conducted the search and selected the appropriate articles by discarding those articles which did not meet the selection criteria, based on title first, abstract second and subsequently using full-text articles. In case of doubt, the article was included in the next round of selection. After full-text selection, the two reviewers compared their selections for consensus. For each article, only those groups of subjects that were treated with a robotic device were included. Since some studies compared several experimental groups that differed by subject type, device used or experimental protocol, the number of groups did not match the number of articles. Thus, we refer to number of groups rather than number of studies.

For each group we filled a record in a structured table. Since the outcome of an intervention can be influenced by many factors, such as the initial level of impairment or the frequency and duration of the intervention, this table contains an extensive set of information (presented in Additional file 2): device used; arm segments involved in training; time post-stroke; number of subjects per group; session duration; number of weeks training; number of sessions per week; total therapy duration; modality (−ies) and features of HRI; baseline impairment measured as average Fugl-Meyer score; and clinical outcome in terms of body functions and activity level. Arm segments involved were categorised as one (single arm segment) or more (multiple arm segments) of shoulder, elbow, forearm, wrist and hand, in which forearm represents pro/supination movement at the radio-ulnar joint. Time since stroke was categorised according to Péter et al. [37], considering the acute phase as less than three months post stroke, the sub-acute phase as three to six months post stroke and the chronic phase as more than six months post stroke.

The main focus of this work is on the interaction between the subject and the robot. Table 1 categorises different ways of intervention commonly found in existing robot-mediated therapy (termed as ‘training modalities’ in this work). In active mode, performance arises from subject contribution only, whereas in passive mode the movement is performed by the robot regardless of subject’s response. In assistive modality, both subject and robot contribution affect movement performance. Passive-mirrored mode applies to bimanual devices, when the movement of the affected side is guided based on active performance of the unimpaired side. In active-assisted mode, the subject is performing actively at the beginning of the movement and the robot intervenes only when given conditions are met (e.g. if the target has not been reached within a certain time), leading to systematic success. In corrective mode instead, in such a case the robot would stop the subject to let then reprise active movement. In path guidance mode, the subject is performing actively in the movement direction, and the robot intervention is limited to its orthogonal direction. Finally, in resistive mode, the robot makes the movement more difficult by resisting the movement received from the subject. We categorised each group according to these modalities. Note that some terms refer to the subject status (i.e. “passive” and “active”), others to the robot behaviour (e.g. “resistive”).

**Table 1:** Training modalities in robot-mediated therapy

**Modality**	**Specifications**	**Schema**
**Assistive**	Subject’s voluntary activity is required during the entire movement. Robots can assist either providing weight support or providing forces aiming at task completion.	
**Active**	The robot is being used as a measurement device, without providing force to subject’s limb.	
**Passive**	Robot performs the movement without any account of subject’s activity.	
**Passive-mirrored**	This is for bimanual robots, when the unimpaired limb is used to control the passive movement of the affected side.	
**Active-assistive**	Assistance towards task completion is supplied only when the subject has not been able to perform actively. At this stage, the subject experiences passive movement of the limb.	
**Corrective**	Subject is stopped by the robot when errors (e.g. distance from a desired position) overcome a predefined value and then asked to perform actively again.	
**Path guidance**	Robot guides the subject when deviating from pre-defined trajectory.	
**Resistive**	Robot provides force opposing the movement.	

However, these categories are not specific enough to classify such different interventions. For example, in the case of reaching movements, the assistive modality would refer to both cases where the robot is providing weight support or applying target-oriented forces. The terms commonly describing modalities of robot-mediated therapy (such as *passive*, *active-assisted*, *resistive*) had to be revised to provide more specific definitions in order to proceed with unambiguous classification. We therefore categorised all the modalities in a different way, specifically based on the features of their implementation. To do so, we identified the following specific technical features used to implement a certain modality (termed ‘HRI features’ in this work): passive, passive-mirrored, moving attractor, assistive constant force, triggered assistance, pushing force (in case of delay), EMG-proportional, tunnels, spring-damper guidance, spring and damper against movement. These categories are defined in detail in Table 2. Note that neither training modalities nor features of HRI are mutually exclusive categories, since groups might have been tested with several training modalities, and each modality might involve the presence of more than one HRI feature. We propose the classification of training modalities and HRI features adopted in this work as a framework for classification for future studies. This is an open framework, so that as new modalities or features are developed and tested, specific categories could be added, to be further referred.

**Table 2:** Features of modalities of human robot interaction and their implementation in robot-mediated therapy

**Feature**	**Specification**
**Passive,** **passive-mirrored**	The device is programmed to follow a desired trajectory/force profile with a strong attractor (up to 1000 N/m) towards it. In the case of passive mirrored, the desired input is given by the subject with the unimpaired hand. In some cases, these trajectories can be set by the therapist during a “learning” phase.
**Moving attractor**	In such a case the assistance is lower than in passive control, the robot is still attracted towards a minimum jerk or smooth trajectory but the amount of assistance can be modulated by varying the stiffness that attracts the robot to the trajectory.
**Triggered assistance**	The subject initiates a movement without assistance. The robot observes that the on-going performance if the task is not completed (e.g. time expired) and intervenes taking the full control, as in the passive mode.
**Assistive constant force**	Force oriented towards the target or weight support when movement is against gravity.
**EMG-proportional**	The power of the EMG signal is used to control the actuators.
**Pushing force (in case of delay)**	A force aligned with the movement direction assists the subject only if there is a delay in comparison with a scheduled motion pattern.
**Spring-damper guidance**	Elastic or visco-elastic force fields aim at reducing the lateral displacement from a desired trajectory.
**Tunnels**	These can be displaced within the virtual environment to produce a haptic feedback only if error overcomes a (large) threshold value. A tunnel can be seen like a lateral spring-damper system plus a dead band zone which makes the haptic intervention discrete in time. This particular cueing of errors relates to a corrective strategy.
**Spring against movement**	The device opposes movements through an elastic force-field pulling back to the start position.
**Damper against movement**	The device generates a force opposing the movement based on current velocity. Although this increases the effort of the subject, it also stabilizes the movement by damping oscillations.
**Not clear**	The information in the text (or its references) did not allow classifying the article. As an instance, if the only mention to the physical interaction was “the robot assisted the subjects during the task”, this was considered not clear due to not providing details on the method of assistance.

We related clinical outcome to factors as segments of the arm trained, time since stroke and modalities and HRI features. We assessed clinical outcome as whether reported improvements were statistically significant or not, for each measure. Outcome was considered separately for body functions and structures (e.g., Fugl-Meyer, Modified Ashworth Scale, kinematics) and activities (e.g., Action Research Arm Test, Wolf Motor Function Test, Motor Activity Log), according to ICF definitions [38]. We categorized each outcome measure to either body functions or activities as defined by Sivan et al. [39] and Salter et al. [40-42]. A group was considered to have shown improvement when at least two-thirds of all the outcome measures within a specific category (of either body functions or activity level) had improved significantly.

## Results

**Figure 1:**
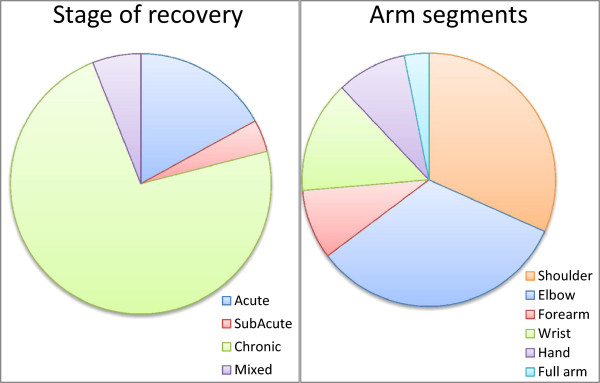
Fraction of groups classified by time since stroke (a) and by segments of the arm trained (b).

In September 2013, our search led to a total of 423 publications. The first two rounds of filtering, based on title and abstract, led to a set of 126 articles. After screening full-text articles, 74 studies were included, with a total of 100 groups treated with robots. Of the 74 studies, 35 were randomised controlled trials and 39 were clinical trials (pre-post measurement), involving 36 different devices. Group sizes ranged from 5 to 116 subjects, with a total of 1456 subjects. Table 3 presents a summarised overview of all included studies, grouped by device. Detailed information for each group is given in the table in Additional file 2: Appendix 2.With respect to stages of stroke recovery (Figure 1a), 73 of the 100 groups included patients in the chronic stage, 17 involved patients in the acute stage, and four groups involved patients in the sub-acute stage. In six cases subjects at different stages of recovery were included in the same group or no information about time since stroke was provided. The average FM score at inclusion among groups of acute subjects was 17.7 ± 12.7 and 25.9 ± 9.5 among chronic subjects. The higher average score for subacute subjects (29.3 ± 7.8) is possibly an outlier due to the small number of observations.

**Table 3:** Overview of included studies: characteristics

**Device**	**Arm segment**	**Phase**	**# of groups**	**References**	**# of subjects (total)**	**Training duration in hours ** **[mean (SD)]**	**Training modalities**	**HRI feature**
MIT-MANUS (InMotion2)	S, E	Acute	6	[43-48]	132	24.2 (10.9)	P, As, AA, PG	SDG, NC, P, PF, TA
		Chronic	20	[48-59]	394	22.3 (17.0)	As, AA, R, Ac	SDG, MA, NC, PF, S, TA
MIT-MANUS (InMotion2)	S, E, F, W, H	Chronic	3	[27,49]	64	24.0 (10.4)	As, AA	SDG, PF
MIT-MANUS (InMotion2 + 3)	S, E, F, W	Chronic	3	[59-61]	63	36.0 (0)	As, AA, Ac	SDG, NC, PF
Bi-Manu-Track	F, W	Acute	2	[62,63]	53	10.0 (0)	P, PM, R	P, PM, S
		Chronic	5	[64-67]	48	26.8 (12.9)	P, PM, R, Ac	NC, P, PM, S
MIME	S, E	Acute	2	[68]	36	12.2 (5.1)	P, PM, AA, R	NC, P, PM,S
		Subacute	3	[69]	24	15.0 (0)	P, PM, AA, R	SDG, P, PM, TA, D
		Chronic	4	[70-72]	37	24.0 (0)	P, PM, AA, PG, R	SDG, P, PM, TA, D
1 DoF robotic device	W	Chronic	1	[73]	8	20.0 (0)	P, AA, A	P, TA
2 DoF robotic device	S, E	Chronic	1	[73]	12	20.0 (0)	P, AA, A	P, TA
3 DoF wrist robotic exoskeleton	F, W	Chronic	1	[74]	9	10.0 (0)	As, Co, Ac	MA,D
5DoF industrial robot	S, E, W	Acute	1	[75]	8	21.4 (0)	P, AA, Ac	P
Amadeo	H	Acute	2	[76,77]	14	9.2 (5.9)	P, AA	P, NC
		Chronic	1	[78]	12	18.0 (0)	AA	NC, P
		Mixed	2	[79]	15	15.0 (0)	P, As, Ac	NC, P
ACT3D	S, E	Subacute	2	[80]	14	Unknown	As, R	ACF
ARM-Guide	S, E	Chronic	1	[81]	10	18.0 (0)	AA	PF, TA
AMES	W, H	Chronic	1	[82]	5	65.0 (0)	P	P
BFIAMT	S, E	Chronic	1	[83]	20	12.0 (0)	PM, C, R	MA, PM, TW
Cyberglove, Cybergrasp + Haptic Master	S, E, W, H	Chronic	1	[84]	12	22.0 (0)	AA	ACF
CYBEX, NORM	W	Chronic	1	[85]	Unknown (27 in total)	Unknown	P	P
PolyJbot	W	Chronic	1	[85]	Unknown (27 in total)	Unknown	AA	EMG
EMG-driven system	E	Chronic	1	[86]	7	30.0 (0)	AA	EMG
	W	Chronic	2	[87,88]	15	42.0 (0)	As, AA, R	EMG, S
	H	Chronic	1	[89]	10	20.0 (0)	As, Ac	EMG
AJB	E	Chronic	1	[90]	6	18.0 (0)	AA	EMG
Hand mentor robot system	W, H	Mixed	1	[91]	10	30.0 (0)	P, AA, Ac	P, TA
Myoelectrically controlled robotic system	E	Chronic	2	[92,93]	14	16.0 (5.7)	As, AA, R	EMG, S
Gentle/S	S, E	Mixed	2	[94]	31	9.0 (6.4)	P, AA, Co, Ac	NC, P
Haptic Knob	F, W, H	Chronic	1	[95]	13	18.0 (0)	P, AA, R	MA, P
HWARD	W, H	Chronic	3	[96,97]	36	22.8 (0.6)	AA, Ac	P, TA
Braccio di Ferro	S, E	Chronic	1	[98]	10	11.3 (0)	P, AA, Co, R, Ac	CF, T,D
MEMOS	S, E	Acute	1	[99]	9	16.0 (0)	P, AA, Ac	P, TA,
		Chronic	3	[99-101]	49	16.0 (0)	P, AA, Ac	P, TA
MEMOS, Braccio di Ferro	S, E	Subacute	1	[102]	20	15.0 (0)	AA, Ac	TA
		Chronic	1	[102]	21	15.0 (0)	AA, Ac	TA
REHAROB	S, E	Mixed	1	[103]	15	10.0 (0)	P	P
NeReBot	S, E, F	Acute	2	[104,105]	28	18.3 (2.4)	P, As	P, PF
REO™ Therapy System	S, E	Acute	1	[106]	10	11.3 (0)	P, As	NC, P
ReoGo™ System	S, E	Chronic	1	[107]	19	15.0 (0)	As, AA, PG, Ac	NC
T-WREX	S, E, W, H	Chronic	2	[108,109]	19	21.0 (4.2)	As, Ac	ACF
Pneu-WREX	S, E, H	Chronic	1	[110]	13	24.0 (0)	As, AA, Ac	ACF, NC
VRROOM, PHANTOM, WREX	S, E	Chronic	1	[111]	26	12.0 (0)		
UL-EX07	S, E, F, W	Chronic	2	[112]	10	18.0 (0)	PM, As	PM, MA, SDG, ACF
BrightArm	S, E, W, H	Chronic	1	[113]	5	12.0 (0)	As	ACF, NC
Linear shoulder robot	S	Chronic	1	[114]	18	Unknown	As, AA	TA,ACF
L-Exos	S, E, F	Chronic	1	[115]	9	18.0 (0)	As, PG	MA, ACF

*Abbreviations:*

Upper extremity segment:

• S = Shoulder

• E = Elbow

• F = Forearm

• W = Wrist

• H = Hand

Human Robot Interactions (HRI):

• P = Passive

• PM = Passive-Mirrored

• S = Spring against movement

• MA = Moving attractor

• TA = Triggered assistance

• PF = Pushing force (in case of delay)

• EMG = EMG-proportional

• T = Tunnels

• SDG = Spring-damper guidance

• ACF = Assistive constant force

• D = Damper against movement

• NC = Not Clear

**Figure 2:**
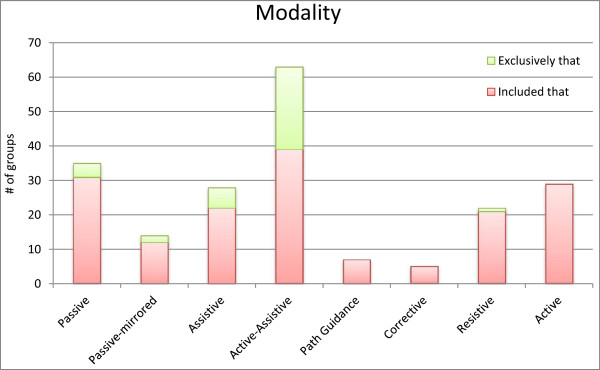
Frequency of each modality among the reviewed groups.

When considering the arm segments (Figure 1b), we observed that training involved shoulder movements for 71 groups, elbow flexion-extension for 74 groups, wrist movements for 32 groups, forearm pronation-supination for 20 groups, and hand movements for 20 groups. Training rarely focused on a single part of the arm, with four groups specifically trained for elbow [86,90,92,93], five groups for wrist [73,85,87,88] and six groups for hand [76-79,116] movements. Training of movements involving the entire upper limb (as those performed during ADL) is not highly recurrent (seven groups) [27,49,84,108,109,113].We then considered the modalities used, and Figure 2 shows an overview of the frequency of usage of each modality. In 63 groups more than one modality was used. Training included active-assistive modality in 63 groups. Twenty-eight groups were trained in assistive modality. Passive training was included in 35 groups. Active and resistive modalities were involved less frequently, in 29 and 22 groups, respectively. The passive-mirrored modality was used in 14 groups, path guidance in seven groups and corrective strategy in five groups.

We also considered these frequencies with respect to the stage of recovery. Passive and passive-mirrored modality are more recurrent for acute than for chronic subjects (with 77 and 24% of the groups of acute trained with these modalities, versus the respective 21 and 11% of chronic subjects). Similarly, modalities more suitable for less impaired subjects as resistive and active are more recurrent among chronic (23 and 30% of the cases, respectively) than within acute subjects (18% for both modalities). Instead, the choice among modalities was not affected by the level of impairment (as measured by FM score). As an instance, subjects trained with passive modality had an average FM of 27.1 ± 12.8 at inclusion versus 23.9 ± 9.6 of those who did not receive this treatment. Subsequently, we considered the HRI features. In 26 groups there was no clear description or reference to the intervention. Passive and passive mirrored modalities showed the same frequency as reported for the previous classification (35 and 14 groups) as the definition of these categories coincides in the two classifications. Triggered assistance, spring-damper along movement and a pushing force followed in order of frequency after passive training (with respectively 26, 18 and 15 groups). Assistance was delivered as a constant force in twelve groups, as a force proportional to the distance from a moving attractor in seven groups and proportional to the EMG activity in eight groups. Resistance was implemented as elastic forces in ten groups and viscous in eight groups. All included studies, except for one, fell in the definitions we provided a priori for categorizations. Recently, a particular paradigm of HRI (error-augmentation) showed clinical benefits [111], but it did not fit in any of the modalities we described a priori. In this modality, the robot tends to displace the subject’s hand from the optimal trajectory by applying a curl force field to the hand. This constitutes a new, different modality, which benefits could be investigated as more studies using it become available.

We then considered the outcome measures, although the positive outcome of an intervention depends on many factors such as the initial level of impairment of the subjects, frequency and duration of the treatment, baseline impairment (for which detailed information is available in Additional file 2: Appendix 2).

Overall, 54 of 99 groups (55%) showed significant improvements in body functions. Twenty-two of the 54 groups who measured outcomes related to the activity level (41%), showed improvements on this level.

With respect to time after stroke and observed that among acute stroke patients, 59% of the groups showed improvements on body functions, and 33% on activity level. In chronic stroke patients, 53% of the groups improved on body functions, and 36% on activity level. For the sub-acute phase, two out of four groups improved on body functions, and two out of three groups improved on activity level.

About outcome for arm segments trained, improvements on body function seemed to be equally distributed between different parts of the arm, but we observed that training of the hand seems to be most effective on the activity levels, with 60% of the groups showing improvements.

Table 4 shows the outcome for different modalities and features of HRI. When relating clinical outcome to specific training modalities, most of the studies (63 of the 100 groups) included multiple modalities in one training protocol. Among them, those including path-guidance (six of the seven groups) and corrective modality (four of the five groups) resulted in the highest percentage of groups improved for body functions (86% and 80%, respectively). However, this may be affected by the limited number of groups. Besides, these groups did not show persistent improvements on activity level. It is noteworthy that the most consistent improvements on activity level were reported for training including the active modality (nine of the 15 groups; 60%).

**Table 4:** Outcomes per training modality and features of HRI

	**% of groups improved at body functions**			**% of groups improved at activity level**		
**Training modality**	*Multiple modalities*	*Only that modality*	*Features of HRI when improved*	*Multiple modalities*	*Only that modality*	*Features of HRI when improved*
Passive	56 (19 of 34)	50 (2 of 4)	P (2 of 2)	44 (10 of 23)	33 (1 of 3)	P (1 of 1)
Passive-mirrored	43 (6 of 14)	0 (0 of 2)		15 (2 of 13)	0 (0 of 2)	
Assistive	57 (16 of 28)	33 (2 of 6)	NC (2 of 2)	38 (6 of 16)	0 (0 of 3)	
Active-assistive	58 (36 of 62)	58 (14 of 24)	TA (5 of 14) EMG (4 of 14) PF + SDG (3 of 14) PF + SDG + NC (1 of 14) CF (1 of 14)	48 (15 of 31)	36 (4 of 11)	TA (2 of 4) CF (1 of 4) NC (1 of 4)
Path guidance	86 (6 of 7)	N/A		50 (2 of 4)	N/A	
Corrective	80 (4 of 5)	N/A		50 (1 of 2)	N/A	
Resistive	64 (14 of 22)	0 (0 of 1)		42 (5 of 12)	N/A	
Active	61 (17 of 28)	N/A		60 (9 of 15)	N/A	
**Feature of HRI**	*Multiple features*	*Only that feature*		*Multiple features*	*Only that feature*	
Passive	56 (19 of 34)	60 (3 of 5)		44 (10 of 23)	50 (2 of 4)	
Passive-mirrored	43 (6 of 14)	0 (0 of 1)		15 (2 of 13)	0 (0 of 1)	
Moving attractor	43 (3 of 7)	0 (0 of 1)		33 (2 of 6)	0 (0 of 1)	
Triggered assistance	60 (15 of 25)	50 (7 of 14)		43 (6 of 14)	44 (4 of 9)	
Assistive Constant force	42 (5 of 12)	20 ( 1 of 5)		17 (1 of 6)	50 (1 of 2)	
Emg-proportional	100 (8 of 8)	100 (5 of 5)		33 (1 of 3)	33 (1 of 3)	
Pushing force (in case of delay)	60 (9 of 15)	N/A		33 (2 of 6)	N/A	
Spring-damper guidance	61 (11 of 18)	N/A		38 (3 of 8)	N/A	
Tunnels or walls	100 (2 of 2)	N/A		0 (0 of 1)	N/A	
Spring against movement	60 (6 of 10)	0 (0 of 1)		20 (1 of 5)	N/A	
Damper against movement	75 (6 of 8)	N/A		60 (3 of 5)	N/A	
Not clear	46 (12 of 26)	50 (5 of 10)		50 (7 of 14)	100 (2 of 2)	

Abbreviations:

Human Robot Interactions (HRI)**:**

P = Passive

PM = Passive-Mirrored

S = Spring against movement

MA = Moving attractor

TA = Triggered assistance

PF = Pushing force (in case of delay)

EMG = EMG-proportional

T = Tunnels

SDG = Spring-damper guidance

ACF = Assistive constant force

D = Damper against movement

NC = Not Clear

The effect of a single training modality (i.e., only one modality applied in a training protocol) was investigated in the remaining 37 groups: 24 groups applied only the active assistive modality, four groups trained with passive movement only, six groups applied only assistive training, two groups with passive mirrored and one group with resistive modality only. The active-assisted modality seemed to have the most consistent impact on improvements in both body functions and activities: 14 of the 24 groups (58%) [43,44,49,60,84,86,88,90,96,100,101] showed significant improvements in body functions. Regarding activities, four of the 11 groups (36%) [43,79,84,96] measuring activity level showed significant improvements after active-assisted training. Training exclusively in passive mode was associated with improvement in body functions for two of four groups (50%) [76,103] and in activities for one of three groups (33%) [103]. With the exclusive assistive modality, two of the six groups (33%) showed significant improvements in body functions [50,61]. In passive-mirrored mode (two groups) and resistive mode (one group), none of the groups showed significant improvements in either body functions or activities.

We also considered whether the inclusion of a modality led to different outcome for subjects at different phases of recovery. Due to the small number of observations for subacute subjects, we neglect those results. Instead, for acute subjects we found that modalities with better outcome on body functions were active (2 out of 3 groups, 67%), assistive (4 out of 6 groups, 67%), active-assistive (5 out of 10 groups, 50%) and passive (6 of 13 groups, 46%). For subjects in acute phase, inclusion of passive mirrored and resistive modality did not lead to improvements in body functions (in none of the 4 and 3 groups, respectively). These results differ from subjects in chronic phase, where inclusion of passive-mirrored modality led to improvement in 75% of the groups (6 out of 8), while the inclusion of resistive modality was effective on 71% of the groups (12 of 17). The path guidance modality led to the best results for chronic patients (6 out of 6 groups improved on body functions). Results for other modalities are similar among them, with all the other modalities being effective on about 60% of the groups. The effectiveness is generally lower on the activity level. For acute subjects, there are not many observations for most of the modalities. Instead, for chronic subjects the inclusion of active modality (62%, 8 out of 13 groups) seemed to perform better than all the others, which were effective in about 40% of the cases. Exclusion to this is the passive-mirrored mode, for which only 1 of the 8 groups (12.5%) improved on activity level.

When we considered the specific HRI features used in the 14 groups who improved on body functions with active-assistance as single modality, five groups used triggered assistance, four groups EMG proportional, four groups a pushing force in combination with spring-damper guidance movement, and one group an assistive constant force. The four groups in active-assisted mode who improved on activity level used triggered assistance (two groups), assistive constant force (one group), and for one group it was not clear which feature of HRI was used.

When considering the clinical outcomes associated with those HRI features within the whole studies reviewed, as for the modalities most of the studies included multiple features of HRI in one training protocol (58 groups). Regarding multiple features of HRI applied in training protocols, those including EMG-proportional, tunnels and damper against movement resulted in the highest percentage of groups improved for body functions (100%, 100% and 75%, respectively). This is followed by spring damper guidance, pushing force, triggered assistance and spring against movement, showing an improvement in body functions in 61% (11 of 18 groups), 60% (9 of 15 groups), 60% (15 of 25 groups) and 60% (6 of 10 groups) respectively. However, the limited number of groups might be affecting this result, especially concerning outcomes at activity level.

The effect of a single feature of HRI (i.e. only one feature of HRI applied in a training protocol) was investigated in the remaining 42 groups. The most common single HRI feature was triggered assistance (TA), which was used in 14 groups. However, only seven of these showed significant improvements in body functions [43,44,96,97,100-102], and in the case of activities, four of the nine groups who measured outcomes on activity level (44%) improved [43,96,97]. The EMG-proportional feature the sole was used in five groups, all showing improved body functions (100%) [85,86,88,90,116], but only one of two groups showed improved activities [116]. A constant assistive force only was used in five groups, of which only one group showed improvements in both body functions and activities [84]. So, when focusing on single features, EMG proportional feature seems most promising, followed by passive and triggered assistance. However, the other HRI features that had good results in combined protocols with multiple features (tunnels, damper against movement, spring damper guidance and pushing force) have not been investigated as single feature of HRI at all. Nevertheless, a common aspect can be derived from the features with most consistent effects, indicating that the active component is promising for improving arm function. For activities, no conclusive answers can be drawn, because of the limited number of studies who have investigated this effect using a single feature at this point. This is also true for analysis of the relation of separate training modalities/HRI features with mediating factors such as initial impairment level, frequency and duration of training, etc.

## Discussion

Our results highlight that robot-therapy has focused mostly on subjects in chronic phase of recovery, while considerably less studies involved subjects in acute and in sub-acute phases (73, 17 and four groups, respectively). However, our results indicate that patients across all stages of recovery can benefit from robot-mediated training.

Despite the evidence that training the hand (alone) is accompanied with improvement of both hand and arm function, we observed that many robot-therapy studies focused on proximal rather than on distal arm training. Also, only a limited number of studies focused on training the complete upper limb involving both proximal and distal arm movements, while there is evidence of benefits for training arm and hand together rather than separately [117]. Additionally, it is known that post-stroke training should include exercises that are as “task-specific/functional” as possible to stimulate motor relearning, which further supports inclusion of the hand and with proximal arm training [28,29]. Additionally, to allow proper investigation of the effect of such functional training of both proximal and distal upper extremity simultaneously, outcome measures at activity level have to be addressed specifically, besides measurements on the level of body functions. However, in all but one study [91] outcomes related to body function were measured, but the effects of robotic training on activities were assessed in only 54 of 100 groups. This prevents adequate interpretation of the impact of robotic therapy and associated human robot interactions on functional use of the arm at this point.

When focusing on the modality of interaction, we observed that training protocols only occasionally included only one training modality, which makes it difficult to examine the effect of one specific modality. This also hindered a detailed analysis of separate effects per training modality and especially their relation with mediating factors such as initial impairment level, frequency and duration of training, etc.

Only a limited number of studies aimed at comparing two or more different robotic treatments. The first of those studies hypothesized benefits of inserting phases of resistive training in the therapy protocol [51]. Subjects were assigned to different groups, training with active-assistive modality only, resistive only or both. There were no significant effects from incorporating resistance exercises. Another study [69] compared a bimanual therapy (in passive mirrored mode) with a unimanual protocol which included passive, active-assistive and resistive training. Again, there were no significant differences between groups in terms of clinical scores. In a different study, active-assistive training delivered with an EMG-controlled device showed larger improvements (in Fugl-Meyer, Modified Ashworth Scale and muscle coordination) with respect to passive movement in wrist training [85]. Assistive forces may also be provided as weight support. In this case, subjects benefitted from a progressive decrease of such assistance, along therapy [80].

Even though there are a limited number of studies comparing separate training modalities, the available data indicated that robot-mediated therapy in active assisted mode led most consistently to improvements in arm function. Whether this mode is actually the most effective one cannot be stated at this point due to lack of a standard definitions used by different studies. It is remarkable that the application of two of the least adopted modalities, i.e. path guidance and corrective, did consistently result in improved clinical outcome. Although this effect might be due to the small number of studies which included them, this suggests that one way to be pursued in future research in order to improve the results of robotic rehabilitation is utilising robot’s programmable interaction potentials, rather than just mimicking what a therapist can do (passive, active-assisted, even resistance to some extent). Experimental protocols including more than one modality should also be sought, and the combined effect of different modalities should be investigated. As an instance, patients might switch from passive toward active modality as recovery progresses.

With respect to the specific strategy (HRI feature) for providing assistance when applying active assistive training, the findings from this review indicate that a pushing force in combination with lateral spring damper, or EMG-modulated assistance were associated with consistent improvements in arm function across studies, while triggered assistance showed less consistent improvement. It is suggested that modalities that stress the active nature of an exercise, requiring patients to initiate movements by themselves and keep being challenged in a progressive way throughout training (i.e., taking increases in arm function into account by increasing the level of active participation required during robot-therapy), do show favourable results on body function level.

The training modalities referred mainly to the description given by the authors of the reviewed studies, but in absence of a uniform definition for identifying robot-human contributions, groups often named the mechanisms used according to their preference and understanding of these mechanisms. Attention to the interaction mechanism between a person and robot is sometimes so limited that often authors did not even mention such mechanisms in their publications (this happened for 26 out of 100 groups). Given a commonly accepted categorisation for these modalities, researchers are then able to compare usefulness of different human-robot interaction mechanisms. It is thought that by providing a common-base for interaction, a larger body of evidence can be provided, i.e. via a data-sharing paradigm, to understand the full potential of robot-mediated therapy for improving arm and hand function after stroke. Ultimately, this can support better integration of robot-mediated therapy in day-to-day therapeutic interventions.

About the effectiveness of different modalities, considering that cortical reorganization and outcome after stroke rehabilitation is positively associated with active, repetitive task-specific (i.e., functional) practice [28,29], human robot interactions stressing these features are preferred. In addition, there is a strong computational basis to push towards delivering minimally assistive therapy [118]. In contrast to this, passive movement is very recurrent, as it may provide more severely impaired subjects with the opportunity to practice. If this is the case, one way to improve the outcome of the therapy in this situation is to tailor the exercise to individual needs while stimulating active contribution of the patient as much as possible, rather than passively guiding to systematic success. Nevertheless, due to the large variety and heterogeneity in training modalities applied (i.e. contents of the intervention), it wasn’t possible to draw conclusions about the role of these additional mediating factors per training modality. More research about comparing different training modalities (with only one specific modality per group) is needed to answer more specifically which training modality would result in largest improvements in arm function and activities after robot-mediated upper limb training after stroke.

## Conclusions

Our review shows that most of the literature about robot-mediated therapy for stroke survivors refers to subjects in chronic phase. In the same way, training most frequently targeted the proximal arm.

Regarding the human-robot interaction, there has been poor attention in documenting the control strategy and identifying which strategy provides better results. This is hampered by the ambiguity in definitions of HRI method employed. While each robot would incorporate a lower-level control, interaction between robot and human is often made possible by incorporating an interaction modality, which is not standardised across different studies.

Therefore, we have proposed a categorisation of training modalities (Table 1) and features of human-robot interaction (Table 2) as an open framework to allow groups to identify the type of mechanism used in their studies, allowing better comparison of results for particular training modalities.

Even though only limited studies specifically investigated different control strategies, the present review indicates that robot-therapy in active assisted mode was highly predominant in the available studies and was associated with consistent improvements in arm function. More specifically, the use of HRI features stressing active contribution by the patient (e.g. EMG-modulated control, or a pushing force in combination with spring-damper guidance) in robot-mediated upper limb training after stroke may be beneficial. More research into comparing separate training modalities is needed to identify which specific training modality is associated with best improvements in arm function and activities after robot-mediated upper limb training after stroke.

## Competing interests

The author(s) declare that they have no competing interest.

## Authors’ contributions

FA conceived of the idea to clarify the mechanism of interaction between human and robots. AB and SN contributed equally to data collection and analysis, and writing of the manuscript. AB and SN both reviewed all abstracts and full articles included in this review. AS, JB, GP and FA provided edits and revisions. All authors read and approved the final manuscript.

## Supplementary Material

Additional file 1 SearchStrategy.pdf: this document contains the full list of keywords used for the search. Click here for file

Additional file 2 ReviewedArticles.xlsx: an Excel spreadsheet which contains an entry for each of the groups, with all the aspects treated in this review (subjects group, arm segments, training modality and features of implementation and indicators for clinical outcome) and others as training duration, and the device used. Click here for file
