# Supplementary material for: Training modalities in robot-mediated upper limb rehabilitation in stroke: a framework for classification based on a systematic review
Source: J Neuroeng Rehabil. 2014 Jul 10;11:111. doi: 10.1186/1743-0003-11-111 (PMC4108977; doi:10.1186/1743-0003-11-111)
Supplement: Additional file 1 — SearchStrategy.pdf: this document contains the full list of keywords used for the search. [file 1743-0003-11-111-S1.pdf]

Additional file 1. Search strategy

Disorder

1. "cerebrovascular disorders"[MeSH Terms]
2. CVA
3. stroke
4. "stroke"[MeSH Terms]
5. "hemiplegia"[MeSH Terms]
6. hemipleg*
7. hemipare*
8. cerebrovasc* OR cerebral vascular
9. #1 OR #2 OR #3 OR #4 OR #5 OR #6 OR #7 OR #8

Intervention

10. "robotics"[MeSH Terms]
11. robot*
12. #10 OR #11

Body segment

13. "upper extremity"[MeSH Terms]
14. "arm"[MeSH Terms]
15. upper extremity
16. upper extremities
17. arm
18. arms
19. upper limb
20. upper limbs
21. shoulder
22. elbow
23. wrist
24. hand
25. #13 OR #14 OR #15 OR #16 OR #17 OR #18 OR #19 OR #20 OR #21 OR #22 OR #23 OR #24

Combination cerebrovascular disorders, robotics & upper extremity

26. #9 AND #12 AND #25

Limitations

27. training
28. therapy
29. #27 OR #28

30. motor
31. function
32. #30 OR #31

Total search strategy

33. #26 AND #29 AND #32
